# Alzheimer’s genes in microglia: a risk worth investigating

**DOI:** 10.1186/s13024-023-00679-4

**Published:** 2023-11-20

**Authors:** Ari Sudwarts, Gopal Thinakaran

**Affiliations:** 1 Byrd Alzheimer’s Center and Research Institute, University of South Florida, Tampa, FL 33613 USA; 2 Department of Molecular Medicine, Morsani College of Medicine, University of South Florida, Tampa, FL 33612 USA

## Abstract

Despite expressing many key risk genes, the role of microglia in late-onset Alzheimer’s disease pathophysiology is somewhat ambiguous, with various phenotypes reported to be either harmful or protective. Herein, we review some key findings from clinical and animal model investigations, discussing the role of microglial genetics in mediating perturbations from homeostasis. We note that impairment to protective phenotypes may include prolonged or insufficient microglial activation, resulting in dysregulated metabolomic (notably lipid-related) processes, compounded by age-related inflexibility in dynamic responses. Insufficiencies of mouse genetics and aggressive transgenic modelling imply severe limitations in applying current methodologies for aetiological investigations. Despite the shortcomings, widely used amyloidosis and tauopathy models of the disease have proven invaluable in dissecting microglial functional responses to AD pathophysiology. Some recent advances have brought modelling tools closer to human genetics, increasing the validity of both aetiological and translational endeavours.

## Background

The first descriptions of microgliosis (termed ‘rod cells’ or ‘granule cells’) were made in the 19^th^ and early 20^th^ Centuries by Rudolf Virchow, Franz Nissl, and Alois Alzheimer (amongst others; see [1]). In a series of publications in 1919, Pío del Río-Hortega identified that these cells are a microglial phenotype, which transition during injury, describing morphological changes and key functions and hypothesising their mesodermal origin (see [1]). Notably, in his initial description of the pathology that adopted his name, Alois Alzheimer described aberrant glial phenotypes [2]. However, research into Alzheimer’s disease (AD) pathogenesis focussed primarily on neurons for over 100 years after this observation.

Within the past two decades, genome-wide association study (GWAS) findings have identified key genetic risk variants for late-onset AD (LOAD) that are expressed exclusively or highly in microglia [3–7]. Initial studies of microglial biology using mouse models of amyloid pathology reported reductions in amyloid burden and preservation of synapse-associated extracellular matrix (perineuronal net) following microglial depletion. However, the amelioration of amyloid pathology was associated with reduced compaction of AD-associated amyloid-β (Aβ) deposits, resulting in increased diffuse plaques and dystrophic neurites [8]. Similarly, genetic methods of targeted microglial depletion have been reported to increase the size of Aβ deposits [9], strengthening the suggestion of a protective microglial function in limiting or compacting Aβ aggregates. Thus, interpreting microglial modulation of AD pathophysiology requires a holistic assessment of pathological consequences at multiple levels rather than isolated readouts. 

Recent advances in single-cell RNA sequencing (scRNAseq) have allowed extensive transcript phenotyping of brain cells, uncovering key gene signatures of microglia in AD pathology. Notably, disease-associated microglia (DAM) was described as a responsive phenotype to amyloid pathology in the 5XFAD mouse model, with key features validated in human AD brains [10, 11]. These plaque-localised cells are characterised by a two-stage activation programme, culminating in TREM2-dependent phenotypic expression of CD11c and LPL (amongst others). Interestingly, the first stage of DAM transition is negatively regulated by BACE1 [12], demonstrating the unique glial cell-type specific functions of this enzyme, despite a clearly established mechanistic role in Alzheimer’s amyloid pathology as the major secretase responsible for amyloidogenic processing of amyloid precursor protein in neurons. 

Other scRNAseq and single-nucleus RNAseq (snRNAseq) analyses have uncovered extensive characterisation of unique cellular states in microglial subpopulations, which transition in response to pathology. Amyloid-responsive microglia (ARM) was characterised by the expression of CD163 [13]. Reactive microglia in CK-p25 mice (which overexpress the cyclin-dependent kinase 5 cleavage product p25 in the postnatal forebrain, triggering AD-like neurodegeneration, atrophy, gliosis, and phosphorylation of endogenous tau [14]) have been grouped into type I and type II interferon-responsive phenotypes [15]. Microglial NF-κB signalling was identified as a central mediator of tau pathology in PS19 mice [16] (however, the failure to use appropriate *Cx3cr1*^*CreERT2*^ mice as *Cx3cr1* haploinsufficiency controls [see below] in this study raises scepticism about its conclusions). A white-matter-specific signature has been reported in microglia from aged mouse brains, which again is dependent on TREM2 [17]. However, the commonalities of these various transcriptomic datasets have led to speculation that they, in fact, describe the same phenotype, with subtle differences emerging from inconsistent clustering algorithms [18]. Indeed, the homogeneity of activation signatures in these heterogenous cells does not appear to reflect the huge differences in pathological insults implemented in identifying them. 

Additionally, there is growing evidence that single-cell transcriptome datasets identified in mouse models of AD-related pathogenesis and neurodegeneration offer poor insight into microglial responses in human AD [19–21]. Whilst post-mortem interval periods may confound the dynamic microglial responses reported in human samples, the validity of modelling slow, age-related diseases with aggressive, transgene-induced pathology requires unbiased evaluation. Indeed, microglia from human AD brains have been reportedly more aligned with the IRF8-reactive phenotype, characterised by increased expression of several homeostatic genes, notably *TMEM119*, *CX3CR1*, and *P2RY12* [21]. Further, the finding that cell-specific transcriptional responses are most dramatic in the early stages of AD may suggest age-related differences in cellular flexibility, implying that modelling this disease in relatively young rodents has certain drawbacks. Thus, shifting towards knock-in models with later disease onset and slower progression may offer more efficacy in both aetiological and translational endeavours. It is also worth noting that half of mouse microglia survive the animal’s lifespan, and proliferation is three times higher during amyloid pathology [22]. In contrast, the human brain microglia may last two decades [23], which is only a fraction of the human lifespan. Therefore, whilst mice present a short-lived model of human disease, their microglia are old relative to human turnover rates. 

## Microglial genes in AD - risk versus prevalence

### High-risk and low prevalence

An obvious disparity exists between the significance and prevalence of genetic AD risk among the ~75 risk loci that have been identified [24, 25]. Most notably, mutations in three genes (*APP*, *PSEN1*, *PSEN2*), which cause early-onset AD (EOAD), are extremely rare in populations. These three genes are ubiquitously expressed and play important functions in neurons and other cell types. As pathogenic mutations are found in only 5% of EOAD patients [26], one may hypothesise the involvement of gene-gene or gene-environment interactions in this aggressive manifestation of AD pathology. *APOE ε4* decreases the age of symptom onset in LOAD but interestingly delays symptoms in EOAD [27]. Additionally, the *APOE ε3* R136S (Christchurch) variant (*APOE*-Ch) has been found to protect against an EOAD *PSEN1* mutation, delaying cognitive impairment and reducing tau pathology despite high levels of Aβ [28, 29]. Similarly, a rare variant in *RELN* (H3447R) has recently been associated with delayed onset of EOAD symptoms in an individual with reduced tau pathology and high levels of Aβ [30]. In mouse models, the loss of *Reln* accelerates both amyloid and tau pathologies [31, 32], suggesting that the protective H3447R variant may increase reelin function. However, this has yet to be determined experimentally. Further, atypical presentations of AD – predominantly affecting visual, language, and motor functions, amongst others – often have early onset (<65 years) and aggressive progressions [33–35]. Knowledge of the risk factors for these relatively uncommon conditions is so sparse that they are grouped with AD based primarily on behavioural symptoms (despite divergent progression patterns). Whilst EOAD is not the focus of this review, current knowledge of microglial involvement in atypical variants will be addressed later.

**Fig. 1:**
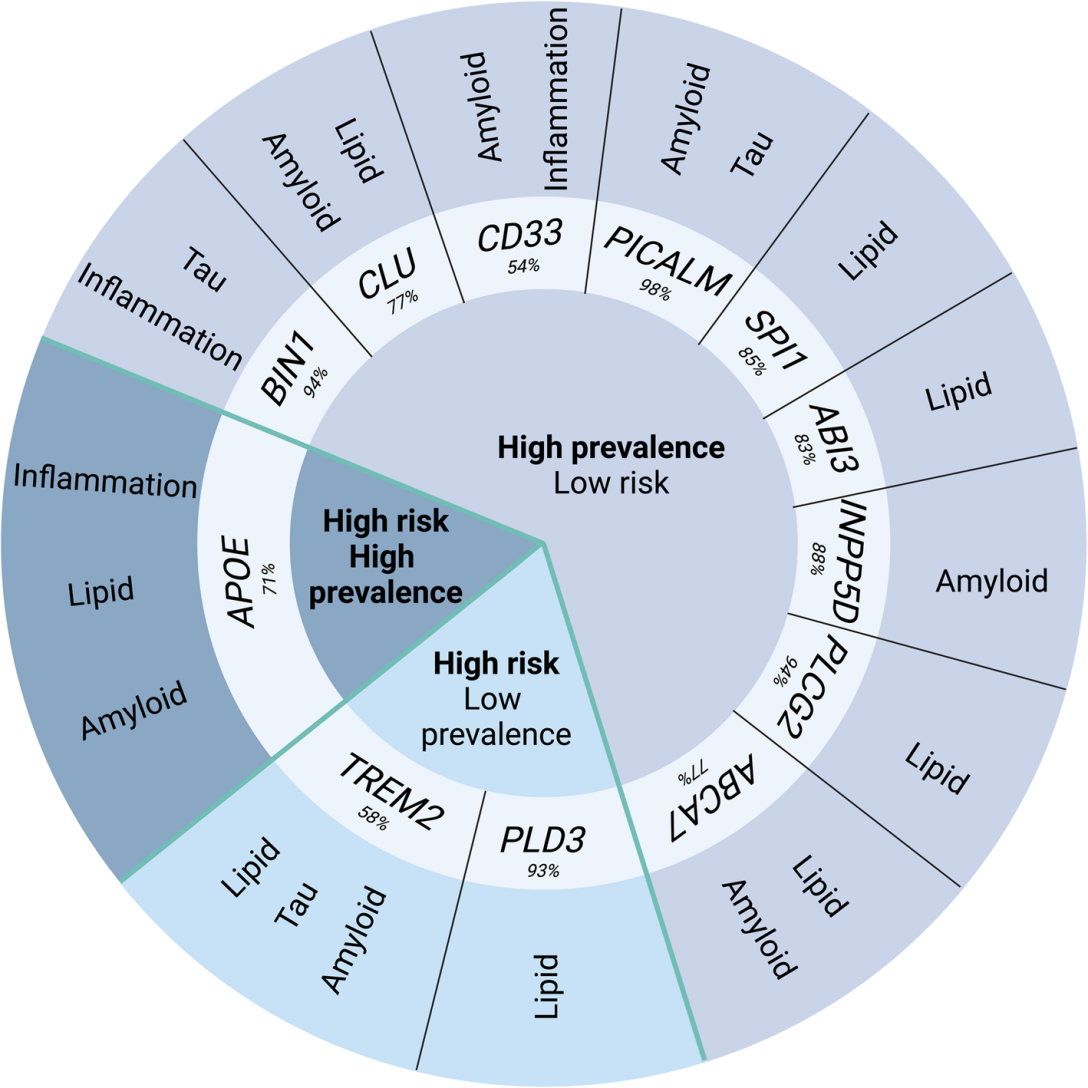
Microglial protein functions in Alzheimer’s disease pathology. Circular representation of microglial genes stratified based on their Alzheimer’s risk and prevalence. The sequence identities between the human and mouse proteins are indicated. The key cellular and pathology-related functions associated with each protein in Alzheimer’s pathology are annotated. Created with BioRender.com

**Fig. 2:**
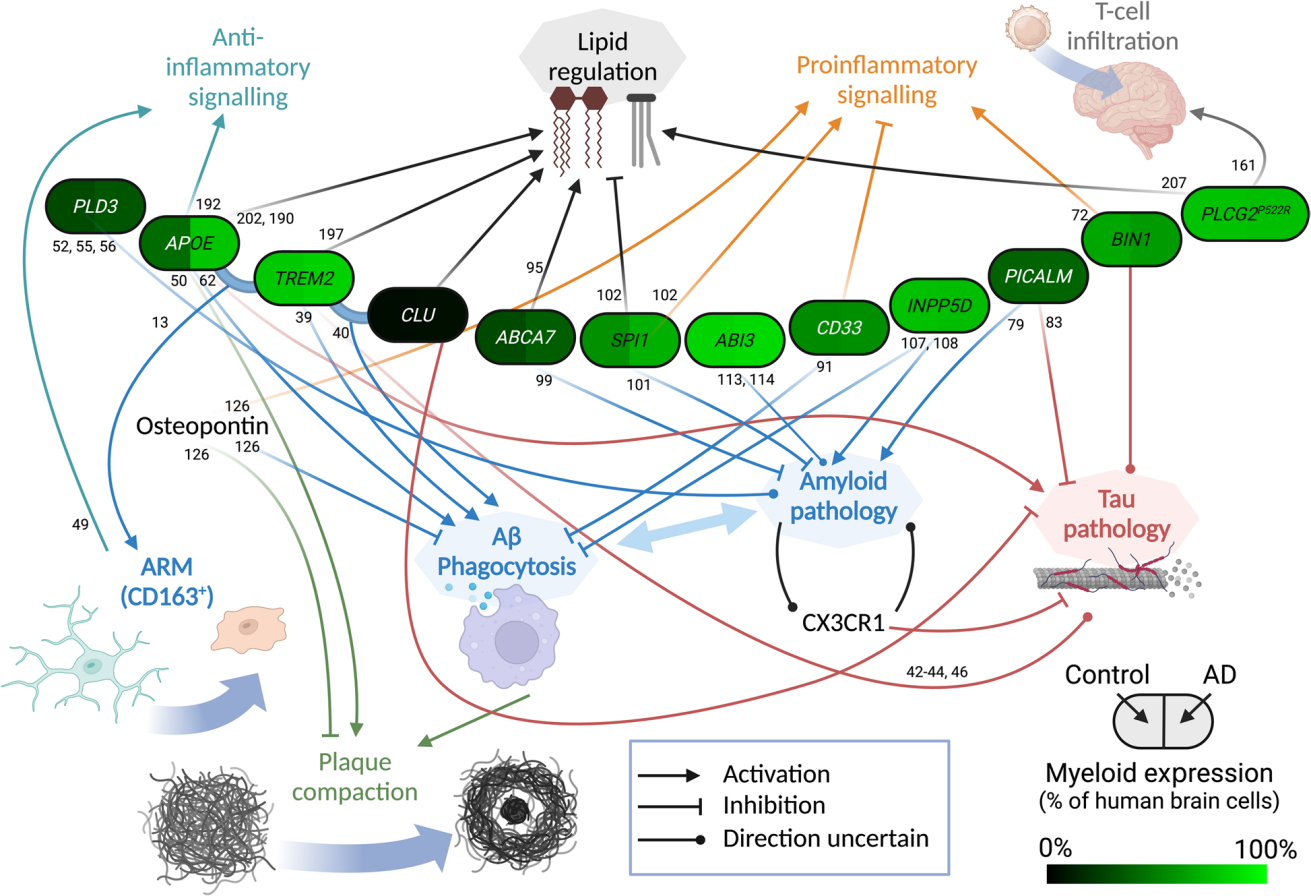
Detailed schematic of microglial protein functions in Alzheimer’s disease pathology. The relationships between key microglial gene products with pathology-related pathways affected in Alzheimer’s disease are indicated. The colour shading of protein names indicates the abundance of the corresponding transcripts in human myeloid cells relative to all brain cells (quantified by single-cell RNAseq [36]). Numbers indicate cited references that support the functional associations depicted in the schematics. Created with BioRender.com

Many genes identified in recent years by GWAS underscore the involvement of microglia-specific functions in AD pathophysiology (Fig. 1). Rare variants that confer a high risk for LOAD include *TREM2* and *PLD3*. These three genes play a role in lipid regulation but have well-established functions specific to AD proteinopathies (Fig. 2).

The microglial surface receptor TREM2 is critical for microglial signal transduction. It regulates a protective microglial response to amyloid deposition in 5XFAD [37] and APP/PS2 [38] mice. Whilst TREM2 binds directly to oligomeric Aβ and facilitates degradation [39], the efficiency of Aβ internalisation by microglia is augmented when Aβ is complexed to lipoproteins [40]. In addition to its requirement for the phenotypic transition from Stage I to Stage II DAM [10], TREM2 is also necessary for the local proliferation of microglia near amyloid deposits [41]. Therefore, the whole activation programme of microglia appears to be dependent on the TREM2 checkpoint, with dysfunction in this surface receptor leaving microglia insensitive to adverse conditions in the amyloid microenvironment. 

The role of TREM2 in tauopathy is less clear, with evidence that it both ameliorates [42, 43] and facilitates [44] tau pathology in different models (hTau & AAV-P301L *versus* PS19 transgenic, respectively). These seemingly contradictory findings may demonstrate a biphasic function at different stages of the disease, with TREM2 protective in the early stages of tau pathogenesis but degenerative in more advanced stages. Indeed, a similar early/late pathology biphasic effect for TREM2 has been reported in amyloid pathogenesis [45], which may reflect age-related alterations to microglial functionality. 

A recent study used a combined model of AD-isolated tau seeds injected into 5XFAD mouse brains [46]. Chronic administration of a TREM2-activating antibody increased activation of microglia proximal to plaques. Astoundingly, whilst amyloid burden was unaffected, aspects of tau pathology increased. These data suggest a role for microglial TREM2 in mediating cross-talk between neuronal amyloid and tau pathologies. It also supports our aforementioned hypothesis of a protective role for TREM2 in the early stages of disease (early tau pathology mentioned above), with detrimental consequences in later stages, and suggests a dominance in TREM2 responding to tau over amyloid. In light of age as the strongest risk factor for LOAD, it is worth mentioning that TREM2 deficiency reduced neuronal loss in normal older mice and downregulated microglial activation and immune transcripts [47]. TREM2 loss has also been reported to increase the density of dendritic spines and cognition [48]. Together, these data suggest that whilst TREM2 function may be specifically beneficial during the early stages of AD pathology, it has detrimental consequences outside these early responses. 

TREM2 interacts with lipidated APOE, regulating Aβ internalisation by microglia [40]. Deletion of *Apoe* and *Trem2* inhibited microglial transition into an amyloid-responsive phenotype, characterised by expression of CD163 [13]. CD163 has an anti-inflammatory function in peripheral macrophages, scavenging pro-inflammatory ligands (notably haemoglobin) [49]. In a mouse model of atherosclerosis, deficiency of both CD163 and APOE increased macrophage expression of proinflammatory and lipid content and increased plaque size [50]. In human AD brains, CD163-expressing microglia were largely CD68-positive [51], supporting the notion that CD68-expressing microglia are anti-inflammatory. 

*PLD3* encodes phospholipase D3 (PLD3), an endolysosomal ssDNA exonuclease involved in nucleic acid-driven inflammatory signalling. A rare variant in *PLD3* (V232M) reportedly doubles the risk of developing LOAD [52]. *PLD3* transcripts are increased in microglia from human AD and mouse models of amyloid and tau pathologies (APP^*NL-G-F/NL-G-F*^ and rTg4510, respectively) [53]. Whilst *PLD3* function in microglia is yet to be determined, its neuronal expression appears to play a protective role by regulating ER stress, nucleotide signalling, and lysosomal function, which are impaired in the V232M variant [54, 55]. Although the loss of PLD3 or the expression of the V232M variant was found to elevate Aβ production in cultured cells [52, 55], there was no change in Aβ production or deposition in young *Pld3* KO mice [56]. 

*APOE* is perhaps in its own category of high-risk and high prevalence, with the frequency of the ε4 allele between 19.2-36.7% in AD patients and 8.9-19% in control populations [57]. *APOE* codes for a core component of plasma lipoproteins, which function to transport and deliver lipids from one tissue or cell type to another. *APOE* is highly expressed in astrocytes in the healthy human brain, but its expression is dysregulated in human AD brains in a cell-specific manner – it is upregulated in microglia and downregulated in astrocytes [20]. This finding highlights the dangers of inferring cell-specific expression changes from bulk tissue analyses. Microglial APOE facilitates migration towards Aβ deposits and phagocytosis, which is impaired in APOE risk variant ε4 [58]. In APP/PS1 mice, the loss of *Apoe* expression (whole-body knock-out) impaired compaction of amyloid deposits, reduced microglia activation, and increased amyloid deposit-associated neuritic dystrophy [59], producing a similar effect to that of microglial depletion mentioned above [8, 60]. Whilst microglial-specific deletion of *Apoe* in 5XFAD mice increased the size of Aβ deposits, there was no change in total Aβ load [61], again suggesting that plaque compaction may be a vital function of microglia. APOE regulates microglial activation in the PS19 mouse model of tauopathy, which is reported to mechanistically drive degeneration in this model [62]. 

### High prevalence and low-risk

Common gene variants (e.g., *BIN1*, *PICALM*, *CLU*, *CD33*, *ABCA7*, *SPI1*) typically confer a lower risk for developing LOAD (Fig. 1) [63–66]. The *BIN1* locus harbours the second-most significant risk for LOAD [67]. The *BIN1* gene encodes multiple isoforms (at least 9 in the brain) of an adaptor protein expressed in neurons, oligodendrocytes, and microglia [68]. It is clear that in the AD brain, there is a decrease in the abundance of neuronal isoforms and an increase in ubiquitous and glial isoforms, which correspond well with the cellular changes in AD [68]. Correcting for neuronal cell numbers suggests that BIN1 expression is protective against AD [69]. Notably, *BIN1*-related SNPs associated with LOAD did not alter *BIN1* expression levels in the human brain [69]. Neuronal BIN1 expression has been shown to facilitate region-specific tau pathophysiology in PS19 mice [70]. Apparently, it has the opposite effect in cultured neurons (in the absence of extrinsic cues from other CNS cell types; see below), where the loss of BIN1 expression was found to promote cell-to-cell pathology propagation [71]. These findings suggest that isolated cell-autonomous signalling alone does not account for the full repertoire of LOAD genetic risk from BIN1. Microglial BIN1 expression is necessary for proinflammatory phenotypic transition and appears to form a reciprocal regulatory relationship with transcription factors PU.1 (*SPI1*) and IRF1 [72]. A microglia-specific enhancer upstream of *BIN1* was identified in human induced pluripotent stem cell (iPSC)-derived microglia through transposase-accessible chromatin sequencing and chromatin immunoprecipitation sequencing, and its functionality was confirmed by introducing a large deletion [73]. However, whether LOAD GWAS SNPs alter microglial BIN1 expression has yet to be ascertained. Thus, whether BIN1 SNPs influence AD pathophysiology through microglial function remains to be established. Moreover, the precise role of microglial BIN1 in AD-specific pathologies is yet to be determined. 

*PICALM* encodes phosphatidylinositol binding clathrin assembly protein involved in clathrin-mediated endocytosis. As with *BIN1*, *PICALM* is expressed in diverse cell types in the central nervous system (CNS), including endothelial cells, neurons, and microglia [74]. The protein products of both of these genes interact with clathrin (although the microglial BIN1 isoforms lack the clathrin-interacting domain [72, 75]), and interestingly, both were found only to pose a significant risk in the absence of the *APOE* ε4 allele [76]. Conversely, the risk for *PICALM* (and one for *CLU*) on episodic memory was also augmented by *APOE* genotype in LOAD patients [77]. Additionally, a review of *APOE*-associated polygenic risk highlights consistent findings of associations with several LOAD genes [78]. Therefore, a full meta-analysis is required to validate these polygenic risks. Functionally, PICALM has been shown to regulate endocytosis of γ-secretase and Aβ_42_ production [79]. A protective variant of *PICALM* (rs3851179A) increases its gene expression [80], suggesting that PICALM function is generally protective. Concordantly, *Picalm* expression in microglia is induced by anti-inflammatory IL4 treatment [81]. Whilst the loss of PICALM reduced Aβ_42_ production in mouse brains [79] and H4 neuroglioma cells [82], *PICALM* haploinsufficiency has been reported to increase tau pathology in Tg30 mice [83]. Therefore, PICALM function in AD may be biphasic, conferring risk in early stages (Aβ deposition) but protection in more advanced pathology (tau hyperphosphorylation). 

*CLU* codes for the secreted lipid carrier protein clusterin (ApoJ) predominantly expressed in astrocytes. Clusterin has been proposed to contribute to chronic inflammation and neurotoxicity through microglial activation [84]. Genetic ablation studies in the PDAPP model suggest that clusterin promotes fibrillar Aβ deposition [85, 86]. Clusterin directly binds Aβ, and this interaction is not affected by clusterin lipidation [87]. However, lipidation of clusterin allows it to be bound by TREM2, facilitating microglial internalisation of Aβ [40]. Loss of clusterin expression was found to exacerbate tau pathology in an AAV-TauP301L expression-based *in vivo* model, and direct interaction of clusterin with tau (0N4R isoform) was observed to reduce tau filament formation in a cell-free system [88]. However, another study reported that clusterin facilitates tau seeding (2N4R isoform) by stabilising oligomeric tau seeds in a cell-based seeding assay [89]. An intracellular form of clusterin was found to more readily interact with both tau and BIN1 in AD brains [90]. Additional investigations on extracellular tau interaction with clusterin (which is mostly extracellular) and cell-type specific functional characterisations of intracellular interactions between clusterin and tau are needed to understand how rare AD-associated *CLU* variants may modulate later stages of disease progression. 

CD33 is a transmembrane protein that inhibits microglial clearance of Aβ [91]. Its transcript and protein expression levels are higher in the brains of individuals with AD [91], and increased expression was related to greater cognitive decline [69]. The *CD33* minor rs3865444T allele has been reported to protect against AD (compared to the major G allele) [91, 92]. The risk allele rs3865444C increases surface levels of CD33 in peripheral monocytes and increases the inclusion of exon 2 (encoding its extracellular immunoglobulin V-set domain). The protective (minor) rs3865444A allele reduces the inclusion of exon 2 [93]. Interestingly, deletion of the *CD33* gene exon 2 or ablation of *CD33* expression led to increased inflammatory response and phagocytosis of Aβ peptides [94]. 

*ABCA7* codes for a ubiquitously expressed ATP-binding cassette transporter, which transports a range of molecules and compounds (including amino acids, peptides, hormones, and lipids) across the plasma membrane (see [95]). The loss of ABCA7 function has been associated with increased AD risk [96–98]. Targeted deletion of *Abca7* alleles affects brain lipid homeostasis and significantly increases cerebral amyloid burden in APP/PS1 amyloidosis model [99]. However, it is unclear if the increase in amyloid deposition pertains to impaired microglial function. Furthermore, ABCA7 expression in mice is required for adequate microglial inflammatory activation in response to LPS challenge [100]. 

*SPI1* codes for the transcription factor PU.1 – a master regulator of the microglial phenotype [101]. Reduced PU.1 expression has been associated with delayed AD onset [102]. Proinflammatory [lipopolysaccharide (LPS)-stimulated] upregulation of *SPI1* is BIN1-dependent [72], highlighting a regulatory relationship between these two risk genes. *In vitro*, *Spi1* knockdown ameliorates inflammatory responses and oxidative stress and increases lipid metabolism [103]. These data suggest that PU.1-mediated inflammatory response is detrimental in the context of AD pathology. 

*INPP5D* (also known as SHIP-1) is an inositol polyphosphate-5-phosphatase whose expression in microglia increases with the progression of amyloid pathology in LOAD selectively in microglia near Aβ deposits [104]. SHIP-1 expression or function negatively regulates phagocytosis in microglia and peripheral macrophages [105, 106]. In one study, *Inpp5d* haploinsufficiency was found to reduce amyloid pathology in the 5XFAD model [107]. However, additional studies using the 5XFAD or the APP/PS1 model found the inverse effect on amyloid burden when *Inpp5d* expression was ablated in microglia using the *Cx3cr1*^*CreER*^ driver [108, 109]. Microglial *Inpp5d* deficiency increased the density of microglia near Aβ deposits, altered plaque-associated microglial gene expression signature, promoted amyloid encapsulation and engulfment by microglia, and protected against Aβ-induced neuronal dystrophy [108, 109]. One caveat to note in the report by Samuels et al. [109] is that this study compared 5XFAD/*Inpp5d*^ΔMG^ mice with 5XFAD/*Inpp5d*^fl/fl^ controls but failed to consider that these two groups also differ by *Cx3cr1* expression. The *Cx3cr1*^*CreER*^ driver line used as the driver to generate *Inpp5d*^ΔMG^ mice expresses the Cre-ER fusion protein from endogenous *Cx3cr1* promoter/enhancer elements such that the Cre insertion knocks out endogenous CX3CR1 expression from the knock-in allele. As discussed below, the loss of CX3CR1 expression affects microglial responses to the amyloid pathology [110, 111]. Thus, additional studies comparing 5XFAD/*Inpp5d*^ΔMG^ mice with 5XFAD/*Cx3cr1*^*CreER*^ controls or using an alternate microglial driver, such as the TMEM119-Cre^ERT^, are needed to clarify the full extent to which *Inpp5d* function relates to microglial reactivity towards Aβ deposits and cerebral amyloid burden. Collectively, these studies show that whilst INPP5D confers some level of LOAD risk, several key functions of this gene have yet to be identified. 

*ABI3* codes for Abelson interactor family member 3, a protein highly expressed in microglia implicated in endocytosis and phagocytosis. The *ABI3* locus contains an AD risk variant (rs616338) associated with immune responses [112, 113]. *Abi3* deletion in 5XFAD mice increases amyloid burden, reduces microglial localisation to Aβ deposits, and impairs microglial migration and phagocytosis [114]. However, no association was found in human AD patients between the *ABI3* risk variant and amyloid load (or other endophenotypes) [113]. 

## Other microglial genes implicit in Alzheimer’s pathology

In addition to AD risk genes, several genes exclusively expressed in microglia have been implicated in microglial responses to AD pathophysiology (Fig. 1). We have briefly discussed a few of them in this section.

The fractalkine receptor C-X3-C Motif Chemokine Receptor 1, encoded by *CX3CR1*, is specifically expressed by microglia within the human and mouse brain [74, 115]. CX3CR1 is crucial for the clearance of myelin debris (and subsequently remyelination) in cuprizone-treated mice [116]. Independent transcriptomics studies observed reduced *Cx3cr1* expression in homeostatic microglia as an early response to Aβ pathology in 5XFAD mice [10, 11, 21]. However, an increase in CX3CR1 protein levels has been observed in this model [117]. Moreover, *CX3CR1* transcript and protein expression are increased in the human brain cortex of patients with AD [21, 117]. Additionally, CX3CR1 regulates microglial responses to Aβ deposition; however, the direction of effect is divergent at different stages of pathogenesis. In the absence of synaptic loss and cognitive impairment, APP/PS1 (4 months) and R1.40 (24 months) mice display reduced Aβ aggregation and microglial localisation at deposits with the loss of *Cx3cr1* in a gene dose-dependent manner [110]. Interestingly, although *Cx3cr1* ablation in the 5XFAD model resulted in fewer Aβ deposits at 4 months of age, there was an acceleration of Aβ deposition by 6 months, concomitant with dysregulation of microglial activation, impaired phagocytic function, and tau phosphorylation [111]. Tau binds to CX3CR1 to facilitate its phagocytosis by microglia [118], with *Cx3cr1* deletion increasing tau pathology [118, 119]. Conversely, overexpression of CX3CL1 (fractalkine; the CX3CR1 ligand expressed by neurons) ameliorated tau pathology in PS19 mice [120]. Thus, the CX3CL1/CX3CR1 signalling pathway profoundly influences Alzheimer’s Aβ and tau pathogenesis. 

*ITGAX* (integrin subunit alpha X) codes for CD11c – a key marker of activated microglia proximal to Aβ depositions in AD brains and mouse models [10, 121]. Increased expression of CD11c (as well as CD11a and CD11b) was first observed in AD brains over 30 years ago [121]. CD11c dimerises with CD18 (encoded by integrin subunit beta 2; *ITGB2*) to form complement receptor 4 (CR4). The related complement receptor 3 (CD11b/CD18) is the dominant receptor for phagocytosis of opsonised apoptotic cells [122] and pathogens by myeloid and lymphoid cells. In macrophages and dendritic cells, CR4 may play a minor role in phagocytosis, dependent on the substrate, although it is dispensable for pathogen phagocytosis in dendritic cells. There is little known of CR4 functions specific to microglia; however, its functions in peripheral immune cells predict that CR4 may have similar roles in microglia. Given the constitutive expression of CD11b in microglia, the specific upregulation of CD11c in amyloid deposit-associated microglia does not necessarily imply an increase in their phagocytic capacity. However, CD11c is thought to facilitate cell adhesion by directly binding to cell adhesion molecules. Importantly, CR4 has been shown to play a key role in adhesion to fibrinogen [123]; elevated fibrinogen in plasma is related to AD risk [124]. Fibrinogen deposits have been reported in 5XFAD and J20 mouse brains, mediating microglial elimination of dendritic spines [125]. Fibrinogen also directly binds with Aβ, and this interaction promotes the aggregation of fibrinogen and Aβ fibril formation [126]. Thus, CD11c (CR4) expression in AD microglia may feasibly increase their anchoring to dendritic spines and mediate synapse elimination. Interestingly, a recent study found that CD11c^+ve^ microglia are divided into two subtypes by osteopontin expression [127]. Osteopontin (coded by *SPP1*) inhibited the compaction of diffuse plaques, Aβ phagocytosis, and degradation but facilitated the production of TNFα. CD11c^+ve^;osteopontin^+ve^ microglia were associated with increased neurodegeneration and inflammation, whilst CD11c^+ve^;osteopontin^-ve^ microglia were designated anti-inflammatory and neuroprotective [127]. 

It must also be noted that *CR1* (*CD35*) locus contains important AD-related SNPs [63, 64, 66] although it is unclear whether these relate to microglia, as *CR1* expression is low in all brain cells, with no transcript-level change observed in AD [36]. *CR1* encodes a type I membrane protein that controls complement activation. There is some evidence that activation of primary microglia by exposure to LPS or Aβ may increase CR1 protein levels and may modulate phagocytic substrate preference [128]. Still, since the majority of the AD-associated *CR1* SNPs do not localise to exons, how *CR1* influences AD risk is poorly understood. 

*IFITM3* codes for restriction factor interferon-induced transmembrane protein 3, a key regulator of cytokine production during viral infection [129]. IFITM3 in neurons and astrocytes has been shown to modulate the APP-cleaving γ-secretase enzyme [130]. IFITM3 expression is elevated in the cortex of patients with LOAD, and the 5XFAD mouse brain [130], and *IFITM3* gene networks are enriched in hippocampi and entorhinal cortices of AD patients [131]. The loss of IFITM3 expression significantly reduces amyloid burden in the 5XFAD model [130]. Interestingly, *IFITM3* upregulation in microglia during inflammation is dependent on the LOAD risk gene *BIN1* [72]. IFITM3 is important for lysosome acidification [132], suggesting the AD risk conferred by these two genes in microglia relates to proteolytic degradation after phagocytosis rather than uptake itself. 

CLEC7A (Dectin-1) is a C-type lectin pattern recognition receptor, which initiates immune responses to fungal infection mediated through SYK [133]. *CLEC7A* expression is upregulated in microglia associated with Aβ deposits in mouse models and human AD [10, 11, 134]. Additionally, *Clec7a* is increased in microglia sorted from aged mice [135]. In the microglia of 5XFAD mice, CLEC7A reportedly activates SYK, increasing compaction and phagocytosis of Aβ [136]. However, as discussed above, using *Syk*^fl/fl^ animals in this study rather than *Cx3cr1*^*CreER*^ mice (control for *Cx3cr1* haploinsufficiency and induced Cre expression) makes it difficult to infer these findings conclusively as SYK-mediated. 

## Common signalling and functions for Alzheimer’s risk genes

Whilst the data regarding microglial genes in LOAD aetiology is far from clear, there are common indications that impaired activation responses may facilitate or augment pathology in stage-specific manners. Both immunity and lipid metabolism have been highlighted as functions of common LOAD risk genes [7, 25, 137, 138] (Fig. 2). The regulation of communication in these systems largely depends on surface receptors, which account for a noticeable fraction of LOAD risk genes. The following section discusses the roles of inflammation and lipids, their relationship, and how surface receptors may mediate disease risk during pathogenesis. 

### Immune responses

As the primary immune cells of the brain, it is perhaps unsurprising that microglial dysfunction is linked to immune processes. However, the direction of immune dysfunction is uncertain. Initial studies speculated that microglia facilitate Aβ production and aggregation and that plaque-localised inflammatory microglia may attack an otherwise homeostatic CNS, initiating degeneration of neurons [139]. However, more recent findings indicate that microglial immune responses are dependent on several LOAD risk genes [10, 37, 45, 72, 84, 100], implying that loss-of-function variants leave microglia unable to respond to the proinflammatory demands of adverse conditions sufficiently. Whether these challenges result from homeostatic processes (i.e., metabolic waste, cellular debris, ‘normal’ apoptosis), acute challenges to homeostasis (e.g., traumatic brain injury, infection, hypoxia), or chronic dyshomeostasis (e.g., metabolic disorders, peripheral inflammation, pollution, diet) remains to be determined and may be indecipherable using inherently variable population-based samples. In this vein, it is worth noting that antenatal hypoxia causes increased expression of microglial activation genes at 2 months, and this effect is augmented in 5XFAD mice [140]. Thus, the interaction between genetic predisposition and CNS challenges (at least during development) may have a long-term impact on microglial phenotypes. 

The receptor for advanced glycation end products (RAGE) can directly bind Aβ [141, 142]. Microglial overexpression of RAGE has been shown to increase the inflammatory response and Aβ aggregation in transgenic mouse models of amyloid pathology [143], whilst microglia cultured from AD patients’ brains show an exaggerated response to Aβ exposure [144]. Apparently, RAGE mediates the transportation of Aβ from the cell surface to mitochondria, promoting inflammasome formation [145]. 

Peripheral administration of endotoxin (LPS) in wild-type mice replicates much of the microglial proteomic changes seen in 5XFAD mice [146]; however, there is evidence that both proinflammatory and anti-inflammatory cytokines are increased in human AD brains [147]. This implies that modelling amyloid pathology in mice with an aggressive younger onset of pathology may only replicate the proinflammatory responses of microglia in human LOAD and not the reparative programmes. Whether this results from the manner of transgene expression (i.e., overexpression of human cDNAs bearing multiple FAD-linked mutations) or the insufficiencies of endogenous mouse genetics remains to be determined. Genetic variants affecting cytokine expression have been reviewed elsewhere [148]. 

Whilst it is well documented that TLR4 mediates proinflammatory signalling following LPS challenge [149], the role of TLR4 in AD pathophysiology is unclear. It has been observed that the loss of TLR4 function results in blunted microglial activation and increased amyloid pathology in 9-month APP/PS1 mice [150, 151], whilst younger (5-month) mice showed impaired microglial activation but no change in Aβ deposition [151]. However, others have found the TLR4 minor allele (resulting in the D299G mutation), which reduces monocyte inflammatory responses to LPS, is more common in control subjects than patients with LOAD [152]. It is plausible that this discrepancy relates to tau pathology in humans with LOAD but is absent in APP/PS1 mice. Indeed, aggregates of hyperphosphorylated tau activate inflammatory responses via TLR4 [153]. However, a complete elucidation of the role of TLR4 in tau pathology is necessary to understand the role of this receptor at different stages of pathology. 

As discussed above, pathological microglial activation depends on TREM2 function. Interestingly, despite its role in mediating Stage II DAM phenotypic transition, TREM2 is an important mediator of anti-inflammatory signalling in microglia *in vitro* [81]. Moreover, it is worth noting that the direction of differential expression change for several DAM genes can be opposite *in vitro* (cultured primary microglia) compared to microglia *in vivo* [72], highlighting the differences between these experimental systems. 

Whilst functional studies into how microglia modulate pathology are only starting to emerge, there are consistent data that surface receptor expression or localisation is integral. As discussed above, several risk genes code for transmembrane proteins, including *TREM2*, *ABCA7*, and *CD33*. Many surface proteins (CD11c, CLEC7A, TREM2, AXL, B2M, CD9) were identified as ‘markers’ of the DAM phenotype [10]. BIN1 facilitates the surface localisation of CD11c without impacting transcript levels [72]. TREM2 glycosylation, affected by AD-associated variant R47H, facilitates its localisation at the cell surface [154, 155]. Interestingly, in addition to these findings, there is evidence that other microglial surface receptors are involved in pathology, either in harmful or protective mechanisms. As a possible important dysregulation in AD pathology, the mis-localisation of surface receptors warrants in-depth investigation. Indeed, microglial receptors present an exciting area for future research (particularly as drug targets in pharmaceutical lead identification); however, a more comprehensive understanding of the extent of surface mis-localisation is necessary to comprehend disease risk at a protein level. 

The complement system has already been alluded to, with CR4 (CD11c/CD18) and CD3 (CD11b/CD18) regulating key cell-specific functions in microglia (adhesion and phagocytosis, respectively) and CD11c^+ve^ microglia emerging in response to amyloid pathology. Additionally, CD88, the complement component 5a receptor 1, is upregulated by microglia associated with Aβ deposits [156] and dystrophic p-tau-laden neurites [157]. Its function facilitates pathology in the Tg2576 mouse model of amyloid pathology [158]. In the mouse brain white matter, CD11c-expressing microglia with reduced levels of CD11b have been observed in response to demyelination [159], suggesting a shift from phagocytosis in this phenotype. Indeed, these microglia are antigen-presenting cells that recruit T cells [159]. Activation of CD8^+ve^ T cells has been observed in patients with AD [160], and T cell infiltration has been reported in transgenic mouse models of aggressive amyloid pathology [161]. A protective allele of *PLCG2* induces antigen presentation gene expression in microglia, promoting CNS infiltration of CD8^+ve^ T cells [162]. Functional studies reported that CD8^+ve^ T-cells infiltrating the brain inhibit the proinflammatory activity of microglia and limit amyloid pathology in the 5XFAD model [163], but in the context of tauopathy in the PS19 model, CD8^+ve^ T-cells drive neurodegeneration [164]. Thus, the involvement of peripheral cell infiltration in AD pathology (including the possible involvement of mast cells, as discussed below) is poorly understood and represents an exciting area for future research. 

### Lipid metabolism and signalling

Microglia are ‘metabolically flexible’, readily switching to glutaminolysis and fatty acid oxidation to facilitate surveillance (process motility) in the absence of glucose [165]. However, inflammatory activation shifts metabolic programming in microglia, suppressing efficient oxidative phosphorylation [166, 167] in favour of inefficient glycolysis [166] (known as the Warburg effect). In activated macrophages, glycolysis feeds the biosynthesis of triacylglycerol, which accumulates in lipid droplets (LD) [168]. Hypoxia induces LD formation in macrophages [169], suggesting that this phenotype may emerge as a stress response, although the induction mechanisms need elucidation. 

Alois Alzheimer observed that “many glial cells show adipose saccules” [2]. Recently, LD formation was reported in microglia proximal to amyloid plaques [170]; however, CD11c^+ve^ microglia associated with Aβ plaques upregulate key genes involved in lipid metabolism [10, 171]. Thus, the balance of lipid accumulation and metabolism in pathological contexts requires mechanistic clarification, with microglial responses likely heterogeneous and phenotype-specific. 

The differences in LD composition further highlight the heterogeneous phenomenon of LD formation. Impaired degradation of cholesterol from phagocytosed myelin (in TREM2-deficient microglia) results in the accumulation of cholesteryl esters in LD [172], which are absent from the triacylglycerol-laden LD in ageing microglia [173]. Triacylglycerol containing LD possibly form an energy reserve in ageing cells. Myelin-induced cholesterol storage may serve for activation-induced alterations in dynamic local membrane composition (e.g., lipid raft formation). However, the need for studies to identify the functional roles of cholesterol in inflammatory responses makes it difficult to interpret these findings mechanistically. 

Cholesterol regulates APOE trafficking of Aβ in microglia, with reduced cholesterol promoting trafficking to lysosomes and subsequent degradation [174]. However, whilst investigations of membrane lipids have shed some light on protein processing, it is clear that lipid dysregulation in AD extends beyond the cell-autonomous level. Additionally, whilst microglia do not express the enzymes necessary for cholesterol conversion into progesterone [175], progesterone and derived hormones profoundly affect microglial inflammatory response programmes [176]. Indeed, progesterone reduced proinflammatory stimulation of TNFα, iNOS, NFκB, and mitogen-activated protein kinase (MAPK)-38 in cultured BV2 cells [177]. Similarly, oestrogen reduced MHC-II^+ve^ microglia following stab injury [178], increased microglial phagocytosis of apoptotic cells and fluorescent beads [179, 180], limited inflammatory release of TNFα, NO_2_, and superoxide [179–181], limited inflammatory expression of iNOS and TLR4 [180, 181], and shifted MAPK phosphorylation from p38-MAPK to p42/44-MAPK [180, 181]. The blunting of microglial inflammatory response in this instance appears to be protective, with oestrogen depletion resulting in decreased microglial clearance of Aβ [182]. There is also evidence that oestrogen protects against tau pathology [183, 184], although it’s difficult to attribute these data specifically to microglia. Testosterone also reduced MHC-II^+ve^ microglia following stab injury [178]. The testosterone metabolite *dihydrotestosterone* inhibited microglial secretion of several proinflammatory molecules via inhibition of TLR4 signalling [185]. However, compared to oestrogen studies, investigations into the effect of testosterone on microglial activation are remarkably sparse. 

The progressive loss of specific lipids in ageing and AD brains has been known for decades (see [186]). The cingulate and temporal cortices are particularly vulnerable to age-related changes in lipid composition [187, 188], and these regions show decreased glucose metabolism in *APOE* ɛ4 carriers [189, 190], suggesting that alleles of this lipoprotein may drive region-specificity of dysfunction and pathogenesis. Reciprocally, lipidation of APOE regulates microglial activation, which is impaired in ɛ4 variant (relative to ɛ3) [58]. Microglial-like cells differentiated from *APOE* ɛ4-expressing iPSCs accumulate lipids [191], notably during ageing [173] and in response to neuronal signals [192], demonstrating an allele-specific inflammatory response. As *Apoe* expression is associated with anti-inflammatory phagocytic microglia in mice [193], these data suggest that ɛ4 induces a destructive proinflammatory phenotype. However, the range of specific functions and molecular interactions of each APOE variant is yet to be fully clarified. 

Low-density lipoprotein receptor (LDLR) is a major receptor that binds APOE/lipoproteins to transport cholesterol and triglycerides in the brain and periphery. Although both APOE and LDLR levels are highest in astrocytes, their signalling in microglia regulates LPS-induced inflammatory response [194]. Additionally, whereas LDLR facilitates APOE endocytosis, *Ldlr* deletion increased CSF APOE levels in transgenic human APOE ɛ3 (210%) and ɛ4 (380%), but not ɛ2 mice [195]; however, cortical APOE levels were only subtly changed in ɛ3 mice, suggesting clearance may not holistically explain the CSF finding. Lipidation of APOE affects its conformation, subsequently affecting binding to receptors and to Aβ (reviewed in [196]). Interestingly, deletion of *Apoe* or the overexpression of LDLR protects against tau pathology in PS19 mice by suppressing microglial activation of *Apoe* expression [197]. Thus, inhibiting APOE-LDLR interactions may prove an efficacious therapeutic strategy against LOAD. 

*TREM2* presents another LOAD risk gene with important lipid-specific implications. TREM2 regulates lipid metabolism in peripheral macrophages, with genetic ablation resulting in increased body fat, insulin, and cholesterol [198]. The transition into Stage II DAM phenotype – characterised by the upregulation of lipid-metabolising genes – is TREM2-dependent [10, 11]. However, AD patients with partial loss-of-function *TREM2*-R47H variant express increased levels of genes involved in lipid metabolism [21]. Studies in mouse models have shown that TREM2 binds lipids associated with Aβ [37] – as well as lipid-associated risk factors APOE and CLU [40] – mediating the microglial response to pathology. TREM2 also binds directly to Aβ species; oligomeric Aβ blocks the TREM2-APOE interaction [199], suggesting competitive TREM2 binding. 

*LPL* codes for lipoprotein lipase, which hydrolyses triglycerides [200]. Loss of *Lpl* in microglia impairs lipid uptake, induces a switch to a proinflammatory signature [201] and causes LD accumulation [202]. LPL is upregulated in Aβ plaque-localised microglia, which show internalised Aβ [10] (and also facilitates Aβ phagocytosis in astrocytes [203]). *Abca7* deletion in mice caused dysregulation of ceramides, sphingomyelins, and hexosylceramides in the absence of pathology [204]. ABCA7 is stabilised by HDL-associated lipoproteins to augment phagocytic function [205]. Transcription factor MEF2C, which has a role in fat deposition in the periphery, limits microglial proinflammatory response [206] and is inhibited in 5XFAD mice [207]. Additionally, a weighted co-expression network analysis of gene, lipid, and protein modules in human AD patient datasets found *PLCG2*, *CR1*, *MEF2C*, and *ABCA7* (as well as non-microglial genes *IL34*, *FERMT2,* and *ANKRD31*) to be associated with lipid modules [208]. Cell biology studies are needed to understand how these risk genes modulate microglial lipid homeostasis and immune responses. Interestingly, sphingolipids regulate the neuronal secretion of Aβ-containing exosomes, which are internalised and cleared by microglia [209]. Sphingolipids such as ceramide are known to be enriched in exosomes, and ceramide levels are increased in AD brain [210], suggesting that exosomal release by neurons may present a mechanism for amyloid aggregation. Potential non-pathological functions of Aβ-containing exosomes in extrinsic signalling from neurons to microglia remain to be investigated. 

Leukotrienes are largely synthesised in neurons, but microglia regulate their synthesis through an unknown non-cell autonomous mechanism [211]. Leukotriene B_4_ is synthesised by microglia in response to intracerebral haemorrhage, signalling neutrophil infiltration and autocrine microglial activation [212]. In the 5XFAD model, treatment with the leukotriene receptor antagonist montelukast reduced neuroinflammation and CD8^+ve^ T-cell infiltration and improved cognitive functions. However, a longitudinal study of individuals using leukotriene receptor antagonists found an association with a slower decline in clinical AD progression but observed no impact on memory performance in patients with AD or MCI and cognitively normal individuals [213]. 

Omega-3 polyunsaturated fatty acids reduce the microglial inflammatory response *in vitro*; however, they also increase phagocytosis of Aβ_42_ peptides [214]. This finding implies that Aβ phagocytosis is a function of homeostatic microglia (in much the same way as phagocytosis of cell debris), suggesting that chronic activation may impair amyloid clearance. Interestingly, maternal deficiency of omega-3 (during gestation and lactation) in rodents increased microglial phagocytosis and the destruction of synapses [215]. It is, therefore, possible that the over-activation of microglia causes a shift from phagocytosis of Aβ to the destruction of synapses, and that this change in the target may be modulated by dietary lipid intake. Similarly, resolvins – derivatives of omega-3 fatty acids – reduce the inflammatory upregulation and secretion of cytokines in microglia [216, 217], inducing an IL4-expressing anti-inflammatory phenotype [218]. However, further studies are required to dissect the specific molecular mechanisms that govern phagocytosis substrate preferences. 

### Phagocytosis

Functionally, phagocytosis has been proposed as a mechanism dysregulated by LOAD risk genes [5]. As several LOAD-related risk genes code for phagocytic receptors (TREM2, CD33) or mediators (PLCG2, PILRA) [5], this function is likely involved in pathogenesis at some stage of the disease. Early studies of Aβ phagocytosis by microglia observed poor degradation within phagosomes, fuelling hypotheses that different microglia subtypes are engaged in amyloid deposition *versus* removal [219, 220]. Indeed, early studies considered microglial interactions with amyloid deposits in transgenic mice to be extracellular, with *in vivo* phagocytosis an unlikely phenomenon [221]. However, recent studies have disproved this notion [222, 223] and also revealed the unexpected finding that microglial phagocytosis regulated by TAM receptor tyrosine kinases Axl and Mer facilitated dense-core plaque formation [224]. Thus, phagocytosis may not be a mechanism for Aβ degradation within the microglia. Interestingly, one study reported that physiological levels of soluble Aβ_42_ peptides undergo extracellular degradation by microglia-derived insulin-degrading enzyme and are not phagocytosed by mouse brain microglia both *in vitro* and *in vivo*, [225]. However, these intriguing findings need to be validated to ascertain the significance of this mechanism in AD pathophysiology. 

Mouse brain microglia that had phagocytosed Aβ *in vivo* contained lower levels of the synaptic marker PSD95; however, they had a higher capacity for synaptosome phagocytosis when cultured [223]. Additionally, these cells expressed high levels of HIF1A, hypothesised to mediate transcription of proinflammatory DAM genes (*SPP1*, *CCL3*) in response to NFκB signalling [223]. It must be noted that phagocytosis of apoptotic neurons involves combined specialised efforts by microglia and astrocytes [226]. How AD pathology affects this intricate intercellular relationship is unclear; however, astrocytes have been proposed to compensate for the loss of microglial phagocytosis by undertaking this function [227]. 

### Age

Importantly, age cannot be overlooked as the strongest risk factor for LOAD. Despite the aforementioned differences in microglial lipid homeostasis and LD composition during ageing and amyloid pathology, the proinflammatory state of aged microglia remains a dysfunctional phenotype [173]. Indeed, several ‘DAM genes’ are upregulated in microglia isolated from aged mouse brains, including *Apoe*, *Lpl*, *Spp1*, *Itgax*, and *Clec7a* [135]. Aged microglia display impaired (reparative) response to IL-4 after injury [228]. Live imaging of retinal microglia showed slower process motility and reduced ramifications in aged mice [229]. Additionally, aged microglia did not present hyper-ramified morphology in response to ATP; instead, they became less dynamic by retracting processes (although not full amoeboid phenotype) and displaying a slower morphological transition [229]. Microglia cultured from aged mice secrete higher levels of proinflammatory cytokines (such as IL6 and TNFα) and display impaired phagocytosis of Aβ_42_ [230]. The senescence-associated secretory phenotype of microglia has been described as an irrevocable cell cycle arrest, resulting in the secretion of inflammatory cytokines [231]. This phenotype develops in aged microglia [232], microglia that undergo excessive proliferation during amyloid pathology [233], and microglia that have phagocytosed neurons containing tau aggregates [234]. Removal of senescent microglia reduces age-associated inflammation and cognitive decline [232], whereas early prevention of proliferation appears to offer some protection against amyloid pathogenesis [233]. Whilst these findings demonstrate age-associated impairments in microglial homeostatic and inflammatory functions, whether these impairments play an aetiological role in AD pathophysiology remains largely undetermined. 

## Modelling Alzheimer’s genes in microglia in vivo and in vitro

Whilst mice are the most common animals used as disease models in AD research, it is worth noting that the protein homology of mouse and human amino acid sequences for LOAD risk factors is largely inadequate. This is particularly notable when considering microglia, where one finds less than 70% homology for over half of microglial-enriched proteins (see Fig.1 and [235]). Additionally, transcript profiles of LOAD risk genes in mouse microglia fail to match the human signatures [236]. This implies the insufficiency of murine genetics to fully model human disease, highlighting the necessity for combination modelling to include human cell types. 

There has been a recent surge of interest in adopting methods of human iPSC differentiation into microglial-like cells (iMG). The ability to differentiate multiple brain cell types from the same pluripotent lines has uncovered cell-type-specific enhancer-promoter interactions, including a microglia-specific enhancer for *BIN1* expression [73]. However, inconsistencies in differentiation methodology induce variability in outcomes between groups, such as the finding of iMG cells expressing the neuronal BIN1 isoform 1 [73], which others have reported absent in microglia [72, 75]. Microglial culture conditions, in general, have been shown to have phenotypic effects on these environmentally sensitive cells. For instance, the presence of albumin affects microglia morphology in culture [237], whilst TGF-β1, TGFβ-2, CSF-1, and cholesterol sustain healthy microglia [237, 238]. Interestingly, the expression of several LOAD risk genes in iMG was affected by TGF-β1 supplementation [238], further highlighting the need to standardise differentiation protocols between research groups. 

A few groups have attempted to further replicate human microglia *in situ* by implanting iMG cells into the brains of microglia-deficient mice [239–243] as well as microglia-depleted slice cultures [244]. The xenografted cells (in immunodeficient mice expressing human *CSF1* on a *Rag2*/*Il2rg* deficient background) appear to replicate much of the transcriptional signature of human brain microglia [239–241]. Whilst presenting an exciting alternative to genetically pure mouse models, the full efficacy of this chimeric approach (as well as its cost-effectiveness) has yet to be fully established within basic and translational neurodegeneration research. Indeed, whilst non-autonomous signalling is maintained with this approach, the interspecies nature of this chimeric modelling strategy suggests some limitations in interpreting cell-extrinsic mechanisms. Nonetheless, this presents a vital tool that may prove invaluable in elucidating LOAD genetic risks in translational settings. 

As mentioned earlier, discrepancies between scRNAseq findings in transgenic mouse models and human AD [19] raise doubt about the validity of modelling the slow progression of age-related AD with aggressive transgene expression in young rodents. Additionally, targeted cell-specific gene manipulation in mice commonly relies on Cre-driven recombination systems. Currently, only a few pan-microglial Cre lines are widely used in research. A set of *Cx3cr1*-driven lines was developed by replacing the genomic locus with the *Cre* or *Cre*^*ERT2*^ coding sequences, thereby losing endogenous *Cx3cr1* expression from the modified allele. The aforementioned effects of reduced *Cx3cr1* expression on microglial phenotype and AD pathology demonstrate the limitation of this system. As an alternative, a *Tmem119*-driven line was developed to express Cre without affecting its endogenous expression [245]. However, there have been criticisms of the *Tmem119*^*CreER2*^ driver line that *Tmem119* is not expressed by all microglia and is also expressed by some peripheral cells [246]. Additionally, with one exception, these microglial Cre driver lines are tamoxifen-inducible. In light of the aforementioned effects of oestrogen on AD pathology, the utility of tamoxifen raises a compounding set of complications. Thus, more suitable systems for microglial Cre expression are urgently needed to facilitate reverse-genetic manipulations in this important cell type. 

It must also be noted that mRNA abundance is often a poor predictor of protein levels. For example, a comparison of protein and mRNA abundance in 384 individuals in the Religious Orders Study and the Rush Memory and Aging Project revealed only 37% of the proteins showed minimally significant concordance with transcript levels [247]. Interestingly, the transcripts most highly upregulated during LPS challenge (notably an NFκB gene network) are repressed translationally by ribosomal binding protein SRSF3 [248], which is expressed in all brain cell types. Thus, whilst many studies have characterised transcriptomic changes in both mouse and human microglia, the potential discrepancy between RNA and protein levels in microglial response to pathological challenges has yet to be characterised. 

## Microglial non-autonomous functions

As the ‘sentinels’ of the brain, microglia are extremely sensitive to communication from other cell types; arguably, their primary function is to receive extrinsic signals and respond appropriately. Over the years, numerous studies that used cultured microglial cells focused on identifying cell-intrinsic signalling mechanisms. The recent popularity of scRNAseq has augmented this trend by revealing microglial subtype transcript signatures and phenotypes associated with autonomous cell-specific signalling mechanisms. However, this approach does not elucidate the complexity of the CNS, which is functionally reliant on extrinsic communication between different CNS cell types. There is a growing interest in studying extrinsic communication between CNS cell types by utilising co-culture systems that enable non-autonomous signalling to be investigated whilst maintaining cell-specific resolution at a functional level. 

The most obvious form of extrinsic signalling from microglia is the release of cytokines during inflammatory responses [149, 249] and in response to AD-like pathologies [16, 249, 250]. Microglial TNFα triggers apoptosis of neurons [251]. IL-1β signals leukocyte and monocyte infiltration via endothelial and ventricular cells, respectively, with neurogenesis also mediated by signalling through endothelial cells [252]. Astoundingly, increases in both IL-1β and IL-10 have been reported to precede Aβ deposition in 5XFAD mice [253], suggesting that this may function as an early preventative mechanism, which becomes chronically dysregulated in AD. Additionally, microglia may respond to neurotoxic signals by releasing fragmented mitochondria, causing proinflammatory activation of astrocytes [254]. As mentioned above, there is also evidence that T cell infiltration in amyloid and tau pathology is regulated by microglia, and this extrinsic signalling to peripheral cells modulates the extent of pathology and neurodegeneration [163, 164]. In addition to destructive non-autonomous mechanisms, microglia exert trophic effects on the CNS. Short-term exposure of microglia to low dose Aβ_42_ increases BDNF [255], which facilitates synapse formation [256], although long-term exposure abrogated the effect and induced a proinflammatory response [255]. Additionally, IL-1β activation of astrocytes has been associated with neuroprotection against NMDA-induced excitotoxicity [257]. 

The non-autonomous signalling from other CNS cell types to microglia must also be discussed. For instance, the aforementioned effects of steroid hormones require their synthesis in astrocytes and neurons [175, 258] or by peripheral cells. Glutamate release by stressed neurons activates microglia, reciprocally triggering neuronal apoptosis [251]. Additionally, the entire phenomena of Aβ- and tau-initiated microglial activation essentially comprise neuron-to-microglia extrinsic signals. 

The CX3CR1 receptor (expressed specifically by microglia in the CNS) has only one ligand – fractalkine (CX3CL1) – which is predominantly expressed by neurons, either surface-bound or secreted [259]. Fractalkine is cleaved by metalloproteinases, including ADAM10 [260]. In response to excitotoxic signalling, cleavage occurs hours before the neuron dies [261], indicating that neurons alert microglia to ongoing damage and impending catastrophe. Interestingly, fractalkine can be cleaved from the neuronal membrane by microglial-produced cathepsin S in response to neuronal injury [262], demonstrating the complexity of microglia-neuron intercommunication. CX3CR1 signalling limits inflammatory responses to LPS [263], suggesting the neuronal release of fractalkine initiates a reparative microglial programme. This aligns with the aforementioned finding that CX3CR1 is downregulated during the response to Aβ deposition [10, 11, 21], characterised by exacerbated inflammation [264, 265]. 

IL33 production by astrocytes stimulates microglial phagocytosis of synapses during development [266]. Both IL33 and its receptor (IL1RL1) are expressed in mast cells [267], which have been observed to increase in numbers in AD patients’ brains [268] and readily infiltrate adult rodent brains. Secretion of tryptase by mast cells induces a proinflammatory phenotype in cultured microglia [269]. Intriguingly, TNF increases mast cell production of IL4 [270], implying a feed-forward mechanism of perpetuated proinflammatory signalling between mast cells and microglia. It must also be mentioned that PLX3397 – used to deplete microglia via inhibition of CSF1R– also depletes mast cells by inhibiting c-Kit. Thus, the findings from microglial depletion studies which utilise this molecule must be regarded in the context of non-cell specificity. 

## Atypical manifestations of Alzheimer’s disease pathology

Atypical manifestations of AD include visual, linguistic, and motor impairments [35] and may account for 38% of young-onset AD [271]. Despite the earlier onset, patients presenting with atypical symptoms do not commonly carry the ε4 allele of *APOE* [272]. Indeed, the *APOE* ε3/ε3 genotype has been reported to account for 59% of young-onset AD patients [271]. Posterior cortical atrophy is the most common atypical variant of AD, with a young age of onset [273]. Variants within several microglial-related LOAD genetic loci have been associated with posterior cortical atrophy, including *APOE*/*TOMM40*, *CR1*, *ABCA7*, and *BIN1* [34]. Investigation of how these genes may influence atypical AD pathophysiology would elucidate the region-specific vulnerability of AD pathologies and how microglia impact these regional variations. 

The fact that such little has been elucidated about atypical young-onset AD cases highlights the multi-aetiological nature of AD pathology as a whole. Whilst dramatic effort has been made to identify cell-specific mechanisms in more common AD manifestations, increased clinical assessment and banking of patient samples for genomics research is necessary to make significant progress in understanding the mechanistic role microglia play in atypical AD. 

## Conclusions

Whilst much recent attention has been given to the heterogeneity of microglial phenotypes, the problems posed by this phenomenon are compounded by the non-uniformity of AD progression. In this light, careful consideration must be given to the stage of the disease being modelled, and interpretations limited to the relative temporal progression in human disease. This is especially true of translational endeavours, in which an efficacious therapeutic intervention in early stages may prove destructive at later stages, and *vice versa*. 

The common themes emerging from current knowledge are that (1) several LOAD risk gene variants dysregulate microglial proinflammatory and anti-inflammatory responses, and (2) ‘normal’ lipid metabolism and signalling mechanisms are impaired. The insufficient inflammatory response of LOAD-risk microglia suggests that, whilst inflammation is commonly observed in AD patients, this has perpetuated from an inability to meet ‘homeostatic’ requirements (i.e., clearance of apoptotic cells and metabolic products) or an inability to appropriately respond to an initiating insult (e.g., traumatic injury, transient ischaemic attack, hypoxia). Whilst trends in research have led to variations in findings (sometimes conflictingly so), advances in research tools continue to provide new methodologies for answering the complex questions posed by a heterogeneous cell type in a non-uniform disease.

## Data Availability

Not applicable.

## Abbreviations

AD: Alzheimer’s disease
GWAS: Genome-wide association study
LOAD: Late-onset Alzheimer’s disease
scRNAseq: Single-cell RNA sequencing
snRNAseq: Single-nucleus RNA seq
DAM: Disease-associated microglia
ARM: Amyloid-responsive microglia
EOAD: Early-onset Alzheimer’s disease
CNS: Central nervous system
CR4: Complement receptor 4
CR1: Complement receptor 1
CX3CR1: C-X3-C Motif Chemokine Receptor 1
TAG: Triacylglycerol
LD: Lipid droplets
MAPK: Mitogen-activated protein kinase
iPSC: Induced pluripotent stem cells
LDLR: Low-density lipoprotein receptor
LPS: Lipopolysaccharide
RAGE: Receptor for advanced glycation end products
iMG: Microglial-like cells
